# Supplementary material for: Taxonomic analysis of metagenomic data with kASA
Source: Nucleic Acids Res. 2021 Mar 30;49(12):e68. doi: 10.1093/nar/gkab200 (PMC8266618; doi:10.1093/nar/gkab200)
Supplement: gkab200_Supplemental_Files [file gkab200_supplemental_files.zip › Suppl_D.zip!/OPAL_comparison_new/by_tool/Kraken2/beta_diversity_bc_superkingdom.pdf]

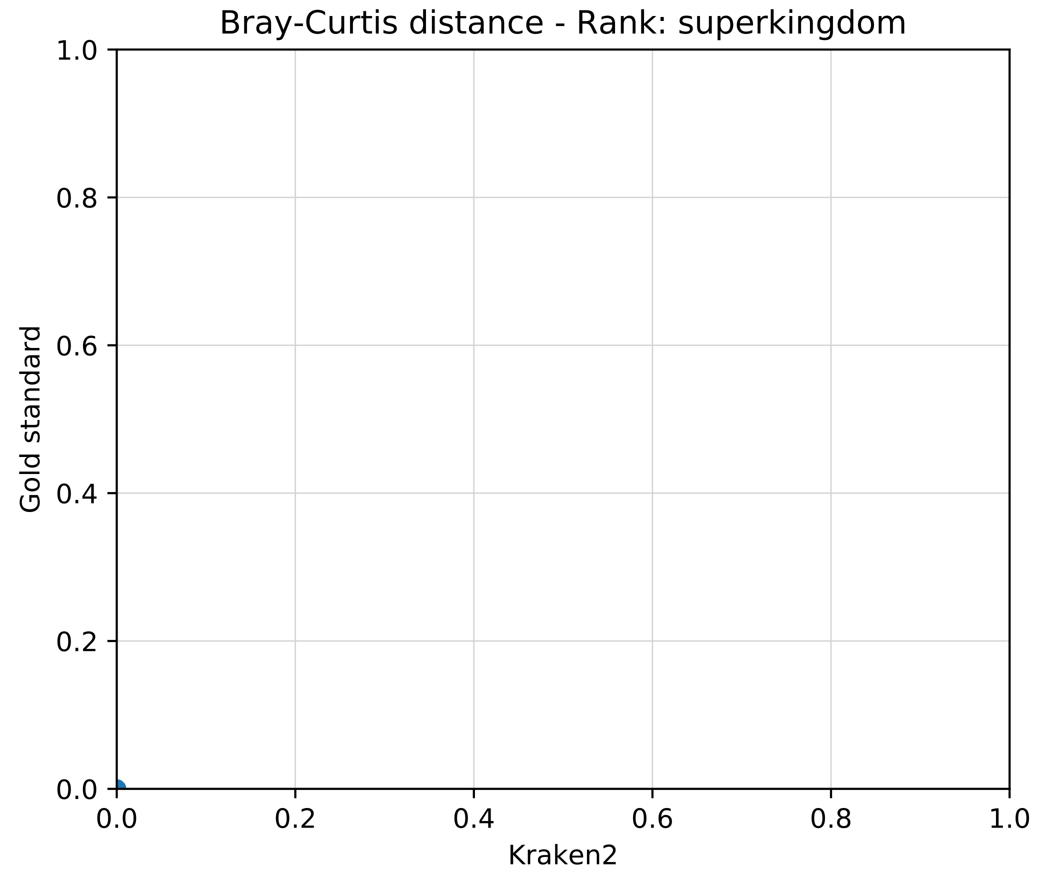
Bray-Curtis distance - Rank: superkingdom
1.0
0.8
0.6
0.4
0.2
0.0
Gold standard
0.0
0.2
0.4
0.6
0.8
1.0
Kraken2
